# *Thiomonas* sp. CB2 is able to degrade urea and promote toxic metal precipitation in acid mine drainage waters supplemented with urea

**DOI:** 10.3389/fmicb.2015.00993

**Published:** 2015-09-28

**Authors:** Julien Farasin, Jérémy Andres, Corinne Casiot, Valérie Barbe, Jacques Faerber, David Halter, Dimitri Heintz, Sandrine Koechler, Didier Lièvremont, Raphael Lugan, Marie Marchal, Frédéric Plewniak, Fabienne Seby, Philippe N. Bertin, Florence Arsène-Ploetze

**Affiliations:** ^1^Laboratoire Génétique Moléculaire, Génomique et Microbiologie, UMR7156, Université de Strasbourg - Centre National de la Recherche Scientifique, Institut de BotaniqueStrasbourg, France; ^2^Laboratoire Hydrosciences Montpellier, UMR 5569, Centre National de la Recherche Scientifique-UM I, UM II, IRD, Université Montpellier 2, CCMSEMontpellier, France; ^3^Laboratoire de Biologie Moléculaire Pour l'Etude des Génomes, CEA-IG-GenoscopeEvry, France; ^4^Institut de Physique et de Chimie des Matériaux de Strasbourg, Université de Strasbourg, CNRS UMR 7504Strasbourg, France; ^5^Plateforme Métabolomique, UPR2357, Centre National de la Recherche Scientifique, Institut de Biologie Moléculaire des Plantes, Institut de BotaniqueStrasbourg, France; ^6^Hélioparc Pau PyrénéesPau, France

**Keywords:** aluminum, arsenic, adaptation, metabolomics, acid stress

## Abstract

The acid mine drainage (AMD) in Carnoulès (France) is characterized by the presence of toxic metals such as arsenic. Several bacterial strains belonging to the *Thiomonas* genus, which were isolated from this AMD, are able to withstand these conditions. Their genomes carry several genomic islands (GEIs), which are known to be potentially advantageous in some particular ecological niches. This study focused on the role of the “urea island” present in the *Thiomonas* CB2 strain, which carry the genes involved in urea degradation processes. First, genomic comparisons showed that the genome of *Thiomonas* sp. CB2, which is able to degrade urea, contains a urea genomic island which is incomplete in the genome of other strains showing no urease activity. The urease activity of *Thiomonas* sp. CB2 enabled this bacterium to maintain a neutral pH in cell cultures *in vitro* and prevented the occurrence of cell death during the growth of the bacterium in a chemically defined medium. In AMD water supplemented with urea, the degradation of urea promotes iron, aluminum and arsenic precipitation. Our data show that *ureC* was expressed *in situ*, which suggests that the ability to degrade urea may be expressed in some *Thiomonas* strains in AMD, and that this urease activity may contribute to their survival in contaminated environments.

## Introduction

Although acid mine drainage (AMD) environments are highly toxic to most living organisms due to the acidic conditions and the presence of elements such as arsenic, a stable microbial community composed of bacteria, archaea, and protists has been known for several years to inhabit the AMD-impacted Reigous creek near Carnoulès (France; Bruneel et al., 2003, 2006, 2011; Duquesne et al., 2003, 2008; Bryan et al., 2009; Halter et al., 2011; Slyemi et al., 2011; Volant et al., 2012). Various *Thiomonas* bacteria isolated from this river were previously characterized, and it has been suggested that by oxidizing arsenite, *Thiomonas* strains may promote the sorption of arsenic by iron oxides and their coprecipitation, resulting in a natural process of arsenic attenuation (Bruneel et al., 2003; Morin et al., 2003; Duquesne et al., 2008; Bryan et al., 2009; Arsène-Ploetze et al., 2010; Egal et al., 2010; Slyemi et al., 2011, 2013). The survival of these *Thiomonas* bacteria probably involves several processes. In particular, metabolic interactions between *Thiomonas* and other microorganisms may be essential to the survival and development of the microbial communities inhabiting the AMD-impacted Carnoulès waters. Interactions of this kind are known to contribute importantly to the survival and development of microbial communities in toxic environments (Jones et al., 2012; Johnson and Amarasekare, 2013). These interactions may be crucial to the functioning of ecosystems and the recycling of chemical elements in toxic environments such as AMDs where the growth conditions are particularly stringent (Wilmes and Bond, 2009). In addition, some of the genes involved in these interactions or in the general adaptation of strains in a particular niche are often found to exist in genomic islands (GEIs). GEIs consist of discrete DNA segments (ranging from 10 to 200 kbp in size), that are present in one species, but are not present in several other related species. These genomic regions sometimes differ in their nucleotide features (G + C content or codon usage) from the rest of the genome, and have often been found to exist in the vicinity of tRNA or tRNA-like genes. The boundaries of these islands frequently correspond to perfect or near-perfect direct repeats (DRs), usually resulting of site-specific integration. These regions often harbor functional or cryptic genes encoding integrases originated from phages or genes involved in plasmid conjugation processes. GEIs, which include elements of other kinds such as integrative and conjugative elements (ICE), conjugative transposons and cryptic or defective prophages, can result from one or several HGT events and genomic rearrangements (Juhas et al., 2009; Bellanger et al., 2014). They can be detected *in silico* by performing genomic comparisons between closely related strains. Studies on these GEIs shed interesting light on the bacterial survival strategies at work in inhospitable environments such as AMD waters.

The pH of the acidic AMD waters in Carnoulès sometimes drops to values of <3. One of the defense mechanisms whereby bacteria resist acidity is based on the degradation of urea: urease catalyzes the hydrolysis of urea, giving ammonia and carbonic acid. In aqueous solutions, the released carbonic acid and the two molecules of ammonia are in equilibrium with their deprotonated and protonated forms, respectively. This reaction increases the pH (Mobley, 2001). A defense mechanism of this kind has been previously described in the pathogen *Helicobacter pylori*, which resists the acidic conditions pertaining in the human stomach (Athmann et al., 2000; Stingl et al., 2002). In sediments collected at the AMD-impacted creek in Carnoulès, urea was detected in the interstitial water (Bertin et al., 2011; Halter et al., 2012). Urea is one of the organic substances produced and excreted by *Euglena mutabilis*, a photosynthesizing bio-indicator of AMD (Brake et al., 2001a,b; Brake and Hasiotis, 2010; Bertin et al., 2011; Halter et al., 2012). Other hitherto uncultivated bacteria such as “*Candidatus* Fodinabacter communificans” (Carn1 and Carn4) may also produce urea in the Carnoulès AMD, since genes involved in the production of urea were detected in their genomes (Bertin et al., 2011). Urea produced by organisms of this kind may then be used by other microorganisms to withstand the acidity. Interestingly, studies on metagenomic sequences have shown that at least one *Thiomonas*-like strain present in this community harbors genes possibly involved in urea degradation processes (Arsène-Ploetze et al., 2010; Bertin et al., 2011).

In this study, we searched for genes involved in urea degradation in the genomes of several *Thiomonas* isolates. Chemical and microscopic tests were first conducted under laboratory conditions in order to determine whether these *Thiomonas* strains are able to degrade urea and if so, whether their urease activity may contribute to acid tolerance and survival. Genes involved in urea degradation processes were found to be present in one GEI in *Thiomonas* sp. CB2. This GEI was incomplete in the other strains tested. The urease activity was found to confer greater viability on *Thiomonas* sp. CB2 in synthetic medium, and to promote metal precipitation in AMD water supplemented with urea. Interestingly, the *Thiomonas* sp. CB2 *ureC* gene was expressed *in situ*, which suggests that urea degradation may favor the survival of *Thiomonas* strains in the Carnoulès AMD.

## Materials and methods

### Bacterial growth conditions

*Thiomonas* spp. CB2, CB1, CB3, CB6, and *Thiomonas arsenitoxydans* 3As were isolated from AMD-impacted water collected at the Reigous creek in Carnoulès (France; Duquesne et al., 2008; Arsène-Ploetze et al., 2010), and *Thiomonas intermedia* K12 was isolated from a corroded concrete wall in the Hamburg sewer system (Milde et al., 1983). These strains were grown in m126 medium: (Na_2_HPO_4_ 4.5 g; KH_2_PO_4_ 1.5 g; Na_2_S_2_O_3_ 5.0 g; NH_4_Cl 0.3; MgSO_4_.7H_2_O 0.1 g; and yeast extract 0.5 g. The pH of the medium was adjusted to 5.0 using H_2_SO_4_). In addition, 4 g.L^−1^ sodium arsenite (from a 500-mM stock solution) were added to the medium when required. The growth potential of *Thiomonas* cells was tested in AMD-impacted Reigous creek water collected on 24 January 2012, which was filtered twice using 0.22 μm filters. Since no urea was detected using a metabolomic approach (see below) in the AMD-impacted water sampled from the creek, urea was added to the AMD-impacted water as specified below. A final urea concentration of 0.02 M (from a 1.67 M stock solution) was adopted because urease activity was detected in the m126 medium when the cells had been previously incubated at this concentration but not at lower concentrations (see the Results Section). Before being incubated in AMD-impacted water, *Thiomonas* strains were grown in the m126 liquid medium. After their growth, the cells were centrifuged for 15 min at 4500 × *g*, washed in 9 g.L^−1^ NaCl and resuspended in filtered AMD-impacted water at the initial Optical Density (OD_600nm_) specified in the figure legends.

### Viability assessment

*Thiomonas* strains were grown in 20 mL of m126 medium at an initial OD_600nm_ of 0.002. Urea from a 1.67 M sterile stock solution was added to obtain a final concentration of 0.02 M. O.D_600nm_ and pH were measured after 2, 3, 5, and 9 days. 500 μL of each cell culture (3As and K12) and 1 mL (CB2) were centrifuged separately at 10,000 × *g* for 10 min and pellets were recovered in 500 μL of sterile NaCl solution (9 g.L^−1^). Viability staining was carried out using a Live/Dead BacLight Bacterial Viability Kit (Invitrogen, Cergy Pontoise, France): 0.5 μL of both SYTO9 green (5 mM) and iodide propidium iodide (1 g.L^−1^) were mixed with 500 μL of cells and incubated for 20 min in the dark. 2.4 μL were used to observe viable or dead cells using a fluorescence microscope (Zeiss Axio-Observer used with a X63 oil immersion lens). Wavelengths of 488 nm and 532 nm were used for SYTO9 and propidium iodide excitation purposes, respectively. Numbers of viable and dead cells were calculated using NIH ImageJ analysis software (http://rsbweb.nih.gov/ij/). These tests were performed in triplicate, except on the 9th day in the case of strains 3As and K12, which were tested in duplicate. When tested in the AMD-impacted water, the mortality rate of *Thiomonas* strains was measured using cell plating methods: cell cultures were diluted in 9 g.L^−1^ NaCl solution and plated onto m126. After 10 days of incubation at 30°C, the colonies were counted.

### Urease activity

The ability of each *Thiomonas* strain to degrade urea was tested with cells grown in liquid m126 media with and without urea. Cells were centrifuged for 15 min at 4500 × *g*, washed and resuspended in PBS buffer (8 g.L^−1^ NaCl, 0.2 g.L^−1^ KCl, 1.44 g.L^−1^ Na_2_HPO_4_, and 0.24 g.L^−1^ KH_2_PO_4_) with the pH adjusted to 5.1, and the volume was adjusted in order to obtain the same number of cells, based on the OD at 600nm. One volume of cell suspension (5 × 10^8^cells) was mixed with 1 volume of BCP reagent (1 g.L^−1^ of Bromo-Cresol Purple with or without 0.83 M urea). In BCP containing urea, the presence of a violet color indicated an increase in the pH due to the urea degradation activity, resulting in the formation of ammonia and carbonic acid. The effects of urea degradation activity on metal precipitation in the AMD-impacted water were assessed by adding 10 U of purified urease (from *Canavalia gladiata* (sword beans), Merck) to 5 mL samples of sterile AMD-impacted water in the presence or absence of 1 g.L^−1^ urea. As a negative control, urease was inactivated before being added to samples by heating it for 5 min at 95°C. Samples were then incubated at 30°C for 24 h and images of the precipitates were taken after a 10-min centrifugation step at 3750 × *g*. Precipitate formation was tested in three independent replicates in AMD-impacted water supplemented or not with urea and active urease.

### Chemical and metabolomic analysis

The AMD-impacted water was filtered at the time of sampling and again at the laboratory and stored at 4°C during the experiments, all of which were completed within 1 year. Due to the characteristics of the creek water (Table 1), Fe(II) precipitated slowly even in non-inoculated water. Abiotic controls were therefore systematically performed and Fe(II) oxidation/precipitation was expressed as the difference between the Fe(II) concentrations measured in the soluble fraction of the non-inoculated and inoculated samples. In addition, the characteristics of the water were determined in 10-month old 0.22 μm filtered AMD-impacted water and compared with those observed at the time of sampling. No significant decrease in the concentrations of elements was detected, except for lead, arsenic and iron, the levels of which decreased by 50, 40, and 10%, respectively, after 10 months and cobalt and barium, the levels of which dropped to below the detection limit in both cases.

**Table 1:** **Chemical parameters of the creek water at the time of sampling**.

**Parameters**	**Values^a^**
pH	3.68
${\text{SO}}_{4}^{2-}$ (mg.L^−1^)	2.991
Fe (mg.L^−1^)	891.50
As (mg.L^−1^)	115.20
Al (mg.L^−1^)	36.95
Ca (mg.L^−1^)	266.80
Mn (mg.L^−1^)	7.41
Zn (mg.L^−1^)	18.53
Co (μg.L^−1^)	249
Ni (μg.L^−1^)	418
Cu (μg.L^−1^)	60.9
Sr (μg.L^−1^)	197
Cd (μg.L^−1^)	85.4
Sb (μg.L^−1^)	<QL
Ba (μg.L^−1^)	6.4
Tl (μg.L^−1^)	287
Pb (μg.L^−1^)	890
U (μg.L^−1^)	20.5

a *Measurements taken 30 m downstream from the source (“COWG”) (Egal et al., 2010). DL, detection limit; QL, quantification limit*.

Concentrations of aluminum, calcium, manganese, iron, nickel, strontium, cadmium, thallium, cobalt, barium, lead, iron, total arsenic, and redox species arsenite (As(III)) and arsenate (As(V)) were determined by performing ICP-MS and HPLC-ICP-MS as previously described (Casiot et al., 2011) or inductively coupled plasma-atomic emission spectroscopy (ICP-AES). As(III) and As(V) species were also observed during metabolomic analysis using previously described GC-MS profiling methods (Lugan et al., 2010). Briefly, samples were vacuum-dried prior to derivatization with methoxyamine hydrochloride (20 mg.mL^−1^ in pyridine) and N,O-Bis(trimethylsilyl) trifluoroacetamide. A mixture of n-alkanes was introduced as retention index standards. The GC-MS system consisted of a 7683 injector, a 6890 gas chromatograph and a 5973 mass spectrometer (Agilent Technologies, Santa clara, USA). Samples were injected twice, in the splitless and split modes (with a split ratio of 50), on an Agilent J&W HP5-MS capillary column with helium as the carrier gas. As(III) and As(V) were identified by comparing the mass spectra obtained with those available in databases (NIST 2008). Total Fe and Fe(II) concentrations were measured using ICP-AES or ICP-MS and the previously described phenanthroline method (Fadrus and Malý, 1975), respectively.

FE-SEM and Energy-Dispersive X-ray Spectroscopy (EDXS, Thermo-Noran Vantage) were performed in order to assess the content of precipitates. In each condition, one pinhead of precipitate was treated with 1 mL hydrogen peroxide in order to eliminate the organic matter, using plastic tubes to prevent metal adsorption. Samples were then centrifuged at 3000 × *g* for 15 min and rinsed with Milli-Q water before performing an additional centrifugation step. Precipitates were then suspended in 100% ethanol. A droplet of the ultra-sonicated suspension was placed on a plasma-treated piece of silicon or carbon wafer providing a smooth, conductive support. After being dried at room temperature, the samples were carbon coated. Standard conditions were used at 12 keV primary beam energy. Since the arsenic L lines at 1.282 keV overlapped with the magnesium K lines at 1.254 keV, control measurements were also performed at 15 keV to check the presence/absence of the arsenic K lines at 10.543 keV. Depending on whether a silicon or carbon wafer was used, the spectra consistently showed a silicon K line at 1.735 keV or a carbon K line at 0.26 keV, respectively, the intensity of which depended on the thickness of the precipitate analyzed. Acquisitions were performed either in the fixed spot mode or by scanning a selected area, and at least three analyses were performed per sample. Due to the topography and the chemically inhomogeneous characteristics of the samples, it was not possible to calculate absolute concentrations in all the samples.

Urea was identified by performing ultra-performance liquid Zic-PiHILIC chromatography coupled to tandem mass spectrometry (UPLC-MS/MS) on an Acquity UPLC system (Waters corp, Milford, USA) coupled to a triple Quadrupole (Quattro Premier XE Waters). Multiple Reaction Monitoring (MRM) methods were used for urea identification purposes at the MS/MS level with the transition: 61 > 44 in the positive Electrospray mode (ESI+). Standard urea was used to determine the chromatographic retention times and mass spectrometry ionization parameters. Absolute quantification was not possible here because the background noise was too large.

### Genomic comparisons

The *Thiomonas arsenitoxydans* 3As genome was previously sequenced and described (Arsène-Ploetze et al., 2010), while the K12 genome was sequenced by the US DOE Joint Genome Institute (NC_014153.1, NC_014154.1, NC_014155.1). The CB2, CB1, CB3, CB6 genomes analyzed in this study were obtained previously (Freel et al., in press) and integrated into the MicroScope platform (Vallenet et al., 2006, 2009, 2013) for analysis. The genome accession numbers of CB2, CB1, CB3 and CB6 (EMBL database) are LK931581-LK931672, LN831666-LN831688, LN831730-LN831775, LN831689-LN831714, respectively. These genomes were compared using the *RGP finder* tool provided with the MaGe platform (http://www.genoscope.cns.fr/agc/microscope/home/) in order to identify GEIs. The *RGP finder* interface can be used to search for potentially horizontally transferred genes (HGT) present in genomic regions (Regions of Genomic Plasticity). The *RGP Finder* method starts by identifying synteny breaks between a query genome and other closely related genomes. It then searches for HGT features (tRNA hotspots, mobility genes) and for the presence of any compositional bias (AlienHunter (Vernikos and Parkhill, 2006), SIGI-HMM (Waack et al., 2006), and GC deviation computation) in the query genome (https://www.genoscope.cns.fr/agc/microscope/compgenomics/genomicIsland.php?act=logout).

### Total RNA extraction from sediment originating from the reigous creek and reverse transcription

The total microbial community was recovered after Nycodenz gradient density separation as previously described (Bertin et al., 2011) from sediments sampled in May 2007 and stored at −80°C. RNA extraction and RT-PCR were performed as described previously (Bertin et al., 2011), with some slight modifications. Cells recovered from sediments were centrifuged at 10,000 × *g* for 15 min at 4°C. Pellets were resuspended in 400 μL of suspension solution [25 mM Tris-HCl (pH 7.6)-10 mM EDTA + 20% glucose (vol/vol)] and transferred into microtubes containing 0.5 g glass beads (0.1 mm in diameter) and 0.5 μL acidic phenol (pH 4.5). Cells were disrupted mechanically with a Retsch's Mixer Mill apparatus (three 30-s cycles of homogenization at maximum speed with 1 min intervals on ice). Microtubes were centrifuged at 13,000 × *g* for 5 min at 4°C. The aqueous phase was placed in a new tube with 1 mL TRIzol reagent (Life Technologies). Microtubes were incubated for 5 min at room temperature. 100 μL of chloroform was added and homogenized by pipetting. The tubes were centrifuged at 13,000 × *g* for 5 min at 4°C. The aqueous phase was then recovered and treated with 200 μL chloroform before being centrifuged at 13,000 × *g* for 5 min at 4°C. The aqueous phase was recovered and purified using the RNeasy Plus Mini kit (Qiagen). RNA integrity was checked by electrophoregram using a BioAnalyser (Agilent) and quantified by measuring *A*_260_ and *A*_280_ with a Nanodrop device. Reverse transcription was performed using the SuperScript® III One-Step RT-PCR System with Platinum® Taq DNA Polymerase (Life Technologies). Each reaction (total volume: 12.5 μL) contained 55 μg total RNA. Thermocycling conditions were as follows: 30 min at 50°C followed by 3.5 min at 94°C, followed by 40 cycles of 15 s at 94°C, 30 s at 55°C and 1 min at 68°C. A final 5-min elongation step at 68°C was added. Primers were designed using three genes: CARN2_aioA_for 5′-CCTGCCATTTCTGCATCG-3′ and CARN2_aioA_rev 5′-GCATTCGGAGTTGTACGC-3′ in the case of the *aioA* gene of the *Thiomonas*-like organisms previously detected at this site (CARN2_0821; Bertin et al., 2011); THICB2v2_ureC_for2 5′-CGAAGGCATGATCCTCAC-3′ and THICB2v2_ureC_rev2 5′-CTCGTCGATGGTGTTGAC-3′ in the case of the *ureC* gene (THICB2V3_370058); THICB2v2_360024_for2 5′-GGGCTGTACACCAACTTC-3′ and THICB2v2_360024_rev2 5′-GCCATCTTCAAGCTGCAG-3′ to amplify the transcript encoding the putative allophanate hydrolase gene (THICB2V3_360023). Negative controls were performed by omitting the reverse transcription step with each pair of primers.

## Results and discussion

### Genes involved in urea transport and degradation are present in one genomic island in some *Thiomonas* strains

**Figure 1:**
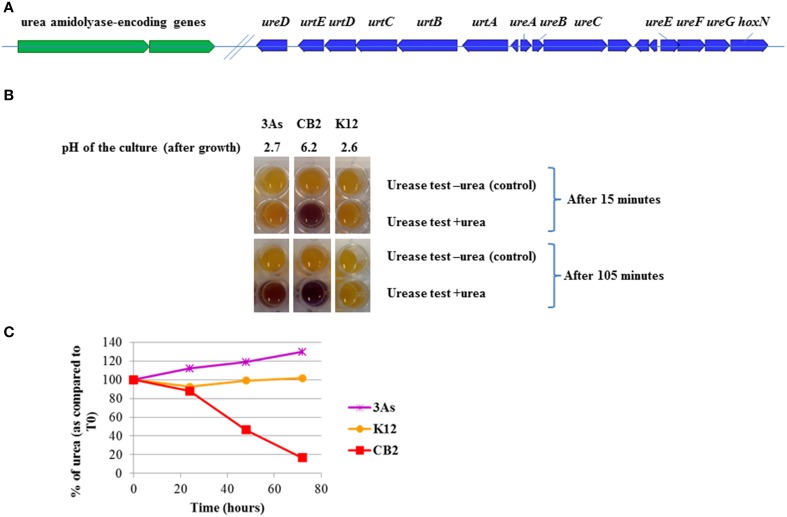
**Genes involved in urea degradation in CB2 and urea degradation activity in ***Thiomonas*** strains. (A)** Genes involved in urea import (*urt*) and degradation (*ure*, in blue or genes encoding the urea amidolyase involved in the UCA-allophanate hydrolase pathway, in green) were detected in a genomic island in the CB2 genome. **(B)** After growth of cell cultures in m126 supplemented with urea (1 g.L^−1^), urea degradation activity was detected using Bromo-cresol purple as described in Materials and Methods. The purple color indicates urea degradation. In the negative control where no urea was added to the BCP reagent, the orange color stands for no activity observed (as indicated by “urease test—urea”). Urea degradation affected the pH of the media when cells were incubated in the synthetic medium m126 supplemented with urea. **(C)** Urea was detected in the the supernatant of CB2, 3As, or K12 cell cultures in synthetic medium m126 supplemented with 1 g.L^−1^ urea, at T_0_ and after 1, 2, or 3 days using mass spectrometry (UPLC-MS/MS). The data are expressed as the percentages of urea (area under the peak) measured at T_0._

The genomes of several closely related *Thiomonas* strains, seven originating from the same AMD (CB1, CB2, CB3, CB6, 3As, ACO3, ACO7) (Freel et al., in press) and one isolated from a sewage pipeline (*Tm. intermedia* K12), have been recently sequenced. A comparative analysis of these genomes using *RGP finder* (see Materials and Methods) showed the presence of 19 GEIs in the *Tm*. sp. CB2 genome (Freel et al., in press). One GEI 88.2 Kb in size (95 CDS) detected using this comparative genomic approach, which is present in the vicinity of another GEI (RGP10; Freel et al., in press) involved in arsenic resistance, was found to have the following characteristics: it is flanked by a miscRNA gene, contains several transposases and shows a compositional bias indicating possible acquisition by HGT (see Supplementary Table 1). Interestingly, this genomic island carries genes involved in urea degradation. In this study, we therefore refer to this GEI as the “urea island.” Two distinct enzymes, urease and urea amidolyase, are known to degrade urea into ammonia and carbonic acid. In bacteria, the urea amidolyase activity results from two separate enzymes, a urea carboxylase and an allophanate hydrolase (Kanamori et al., 2004). The “urea island” in *Tm*. sp. CB2 carries genes encoding these enzymes (*ure* genes encoding urease and two genes which are essential to the urea carboxylase (UCA)-allophanate hydrolase pathway) and *urt* genes involved in urea import (Figure 1A). A second copy of the *urtABCDE* genes was present at another locus in the CB2 genome (from 2246372 to 2251947). This duplication was not detected in the genomes of the other strains tested. The urea island also carries genes involved in metal, sulfur, lipid and nitrogen metabolism, including 27 genes involved in nitrate transport and reduction in particular. It also contains genes encoding ABC transporters and several hydrolases that contribute to the degradation of various compounds such as cyanate and creatinine (Supplementary Table 1).

**Figure 2:**
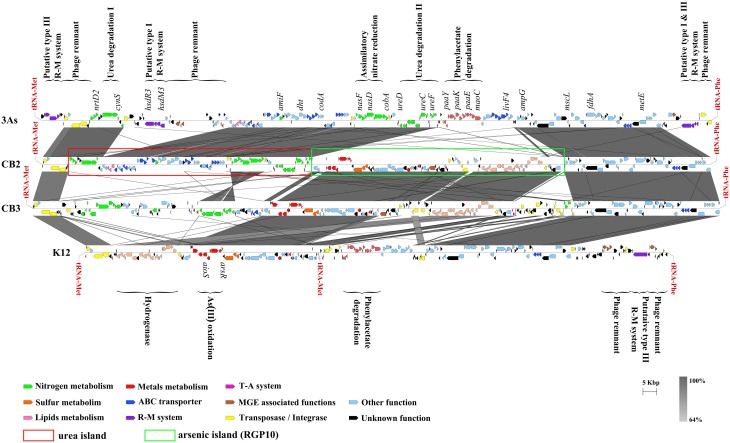
**Comparison between the ***Thiomonas*** genomic regions carrying urea islands**. The large genomic region comprising the urea island and another island, RGP10 in CB2 and the synteny between 3As, K12, CB3, and CB2 genomes are shown. The percentage of nucleotide identity is expressed in shades of gray (see the gray scale). Figures were generated with Easyfig (Sullivan et al., 2011).

**Figure 3:**
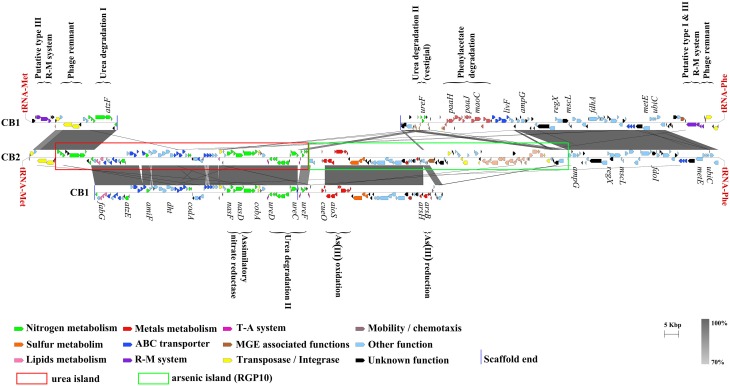
**Comparison between the ***Thiomonas*** genomic regions carrying a urea island**. The large genomic region comprising the urea island and another island, RGP10 in CB2 and the synteny between CB1 and CB2 genomes are shown. The percentage of nucleotide identity is expressed in shades of gray (see the gray scale). Figures were generated with Easyfig (Sullivan et al., 2011).

**Figure 4:**
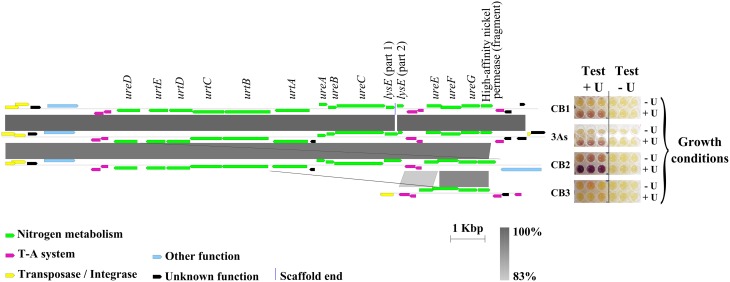
**Comparison between the genes involved in urea degradation and urease activity in the ***Thiomonas*** strains investigated**. Only part of the urea island carrying the *ure* and *urt* genes involved in urea degradation is shown. The figure shows the synteny between CB1, CB2, CB3, and 3As. The percentage of nucleotide identity is expressed in shades of gray (see the gray scale). Figures were generated with Easyfig (Sullivan et al., 2011). The urea degradation activity is shown on the right: cells were grown in the absence (−U) and presence (+U) of urea. After growth, cells were incubated in the presence of urea and Bromo-cresol purple for 30 min, as described in Materials and methods (as indicated by “test + urea”). As a negative control, the test was performed in the absence of urea in the reaction mixture (as indicated by “test − urea”). In the case of the test performed in the presence of urea, the purple color indicates urea degradation.

The synteny of the genes detected in and around the urea island in CB2 was compared with that of the corresponding genes in CB1, 3As, CB3, CB6, and K12 (Figures 2, 3), and these analyses showed that some blocks of genes were highly conserved, although possibly translocated or inverted, whereas others were lacking in some *Thiomonas* strains. In particular, genes involved in urea degradation and transport processes were found to be present in 4 genomes from *Thiomonas* strains (CB2, CB1, CB6, and 3As) but not in CB3 or K12 (Figure 4), while the genes detected in the vicinity of the *ure* genes in CB2 are present in both of these strains (Figure 2). These results suggest that these urea islands have evolved differentially in these closely related *Thiomonas* strains. GEIs may endow bacterial strains with particular abilities (Juhas et al., 2009). Since urea degradation is thought to be involved in acid tolerance in some bacteria such as *H. pylori* (Stingl et al., 2002), and since *Thiomonas* strains were isolated from acidic AMDs, the contribution of these genes to acid tolerance was further investigated.

### Urea degradation activity prevents acidification of the medium and cell death during the growth of *Thiomonas* sp. CB2

First, the urea degradation activity of *Thiomonas* sp. CB2 was tested with cells previously grown in culture media supplemented with various urea concentrations (Supplementary Figure 1). An optimized colorimetric test (see Materials and Methods) showed the occurrence of urea degradation in the case of cells grown with 0.02 M urea but not in those pre-grown with lower urea concentrations (Supplementary Figure 1). These findings suggest that the expression of the corresponding genes is induced in the presence of urea. This urea degradation activity could not be quantified but was reproduced at least five times and confirmed by the results of metabolomic studies (Figures 1B,C). No activity was detected with either *Thiomonas intermedia* K12 or *Thiomonas* sp. CB3 (Figures 1, 4; Supplementary Figure 1), in agreement with the genomic analyses, which showed that these two strains carry no genes involved in urea degradation (Figures 2, 4). The fact that strains 3As, CB1, and CB6 showed very low levels of activity (Figures 1, 4; and data not shown) suggests that under the conditions tested here, the genes involved in urea degradation processes are not strongly expressed in these strains or that the corresponding enzymes are not fully active. Close analysis of the genes detected in the urea islands of these *Thiomonas* genomes showed that two genes differed in CB2 in comparison with 3As, CB1, and CB6: (i) one gene encoding a putative transporter belonging to the *lysE* family was fragmented in 3As; (ii) a gene encoding a high-affinity nickel permease was shorter in CB1, CB6, and 3As than in CB2 (Figure 4, and data not shown). Since nickel plays an important role in urease activity (Stingl et al., 2002), this may explain why the degradation activity was lower in these strains than in CB2. This possible explanation for the difference in urea degradation activities will have to be tested when appropriate genetic tools have been developed for studying these bacteria. All in all, the present findings suggest that several *Thiomonas* strains contain a genomic island harboring genes which are either directly or indirectly involved in the degradation of urea, whereas other strains lack these features, which results in differences between strains in terms of their ability to degrade urea.

**Figure 5:**
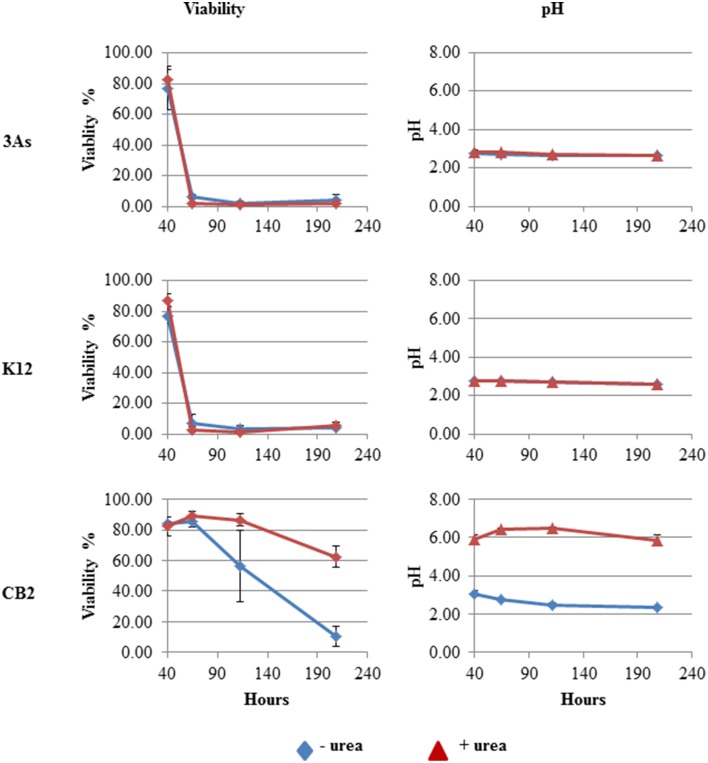
**Effect of urea degradation on cell viability**. Viability and pH were measured during the growth of the *Thiomonas* strains in m126 medium in the absence and presence of urea. Error bars indicate standard deviations based on triplicate cultures.

The effects of urea degradation on acid tolerance were further tested in three strains, CB2, 3As, and K12. During the growth of these three strains in m126 in the absence of urea, the pH of the culture medium dropped to approximately 3.0 or less within 24 h, and stabilized after 50 h (Figure 5). The viability of the cells decreased rapidly, probably due to this pH decrease. In the presence of urea, the pH of the CB2 growth medium remained at a value of approximately 6 because of the production of ammonia and carbonic acid during the urea degradation process, whereas in both K12 and 3As, the pH dropped to 3 even in the presence of urea in the growth medium (Figure 5). CB2 cells were less viable when grown without than with urea (Figure 5). The viability of 3As and K12 was similar whether cells were grown in the presence or absence of urea, as well as being similar to that of CB2 cells grown without any urea (Figure 5). These data show that the urea degradation activity of CB2 in synthetic media contributed to preventing the acidification of the medium during cell growth and enabled CB2 to survive more efficiently than 3As and K12 under the conditions tested here. The acid resistance conferred on CB2 by urease activity may result from a similar mechanism to that found to occur in the human pathogen *H. pylori*, which resists acid in the human stomach by degrading urea into carbonic acid and ammonia, the deprotonated and protonated forms of which are in a state of equilibrium (Athmann et al., 2000). It was then proposed to test whether this activity played a role when this bacterium was incubated under AMD conditions.

### The degradation of urea by *Thiomonas* sp. CB2 promotes the precipitation of the toxic metals in AMD-impacted water supplemented with urea

**Figure 6:**
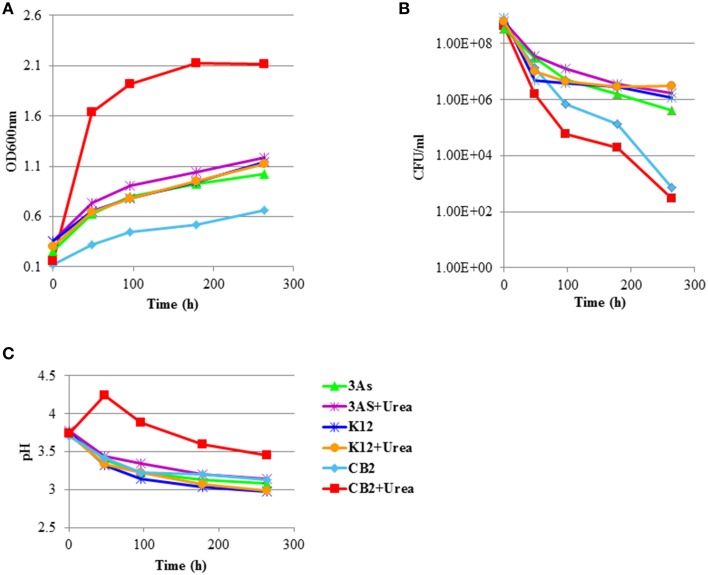
**Effects of incubating ***Thiomonas*** strains in AMD-impacted water. (A)** OD_600nm_, **(B)** Viability expressed in CFU/mL, and **(C)** pH was measured in AMD-impacted water incubated with *Thiomonas* strains in the presence and absence of 1 g.L^−1^ urea. This experiment was representative of three independent experiments.

The effects of the CB2 urea degradation activity were therefore tested in contaminated water from the AMD-impacted Carnoulès creek under laboratory conditions (Table 1). No urea was detected in the AMD-impacted water used in these experiments (data not shown). CB2, 3As, and K12 could therefore be incubated in this AMD-impacted water, whether or not it was supplemented with urea. When no urea was added to the AMD-impacted water, the growth of all three strains, as shown by an increase in the OD_600nm_, resulted in a decrease in the pH (Figures 6A,C). However, when CB2 was incubated in the AMD-impacted water with urea, the pH increased within 48 h (Figure 6C), reflecting the occurrence of urea degradation activity. The number of CFU.mL^−1^ decreased with time, which suggests that the *Thiomonas* cell survival ability decreases under these conditions (Figure 6B). Contrary to what was observed in m126 medium, the viability of the CB2 cells did not increase in the AMD water in the presence of urea, and was even slightly impaired. The urea degradation process therefore did not improve the rate of CB2 survival under these conditions tested. The acid resistance mechanism involving urea degradation activity may not have been sufficiently strong to improve the viability of *Thiomonas* sp. CB2 in the AMD-impacted water, the pH of which was lower than that of the synthetic medium.

**Figure 7:**
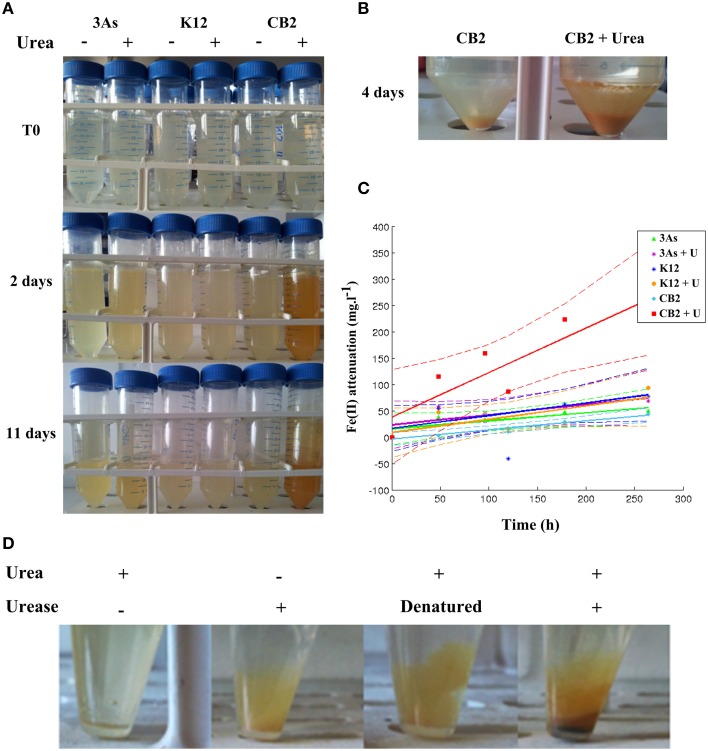
*****Thiomonas*** urea degradation activity promotes Fe(II) oxidation/precipitation in AMD-impacted water supplemented with urea**. K12, 3As, and CB2 were incubated at an initial OD_600nm_ of 0.2 – 0.3 in AMD-impacted water in the absence and presence of 1 g.L^−1^ urea. **(A)** The medium supplemented with urea and CB2 acquired an orange color with time, which did not occur with 3As or K12. **(B)** Orange precipitate detected after centrifuging 4-day cell cultures of CB2 in AMD-impacted water supplemented or not with 1 g.L^−1^ urea. **(C)** Kinetics of Fe(II) oxidation/precipitation. Fe(II) oxidation/precipitation is expressed as the difference between the Fe(II) concentrations measured in the soluble fractions of the non-inoculated and inoculated samples. The dotted line gives the 5% confidence interval of each of the regression lines, computed with the MATLAB fitlm command. **(D)** Effects of urease activity on the formation of orange precipitate in the AMD-impacted water. AMD-impacted water was supplemented with 10 U of purified urease in the presence and absence of 1 g.L^−1^ urea. As an additional control, urease was heat-inactivated for 5 min at 95°C.

An orange precipitate accumulated during the experiments with CB2 in AMD-impacted water in the presence of urea, which was not observed in samples where no urea degradation activity was possible (Figures 7A,B). To test whether this precipitate was correlated with urease activity, purified urease and urea were added to sterile AMD-impacted water. A substantial orange precipitate was observed when both urease and urea were added, whereas no precipitate occurred when only urea was added, and less precipitate when urease was heat-inactivated (Figure 7D). The presence of this orange material suggested that Fe was precipitated when either urease or bacteria with urea degradation activity were present in the AMD water supplemented with urea. A covariance analysis was performed with MATLAB R2014a (using the *aoctool* and *multcompare* commands) on the quantity of soluble Fe(II) measured in the dissolved phase vs. time. This analysis showed that the rate of Fe(II) oxidation and subsequent Fe(III) precipitation was significantly higher (*p* < 0.01) in the case of CB2 grown with urea than in that of the other cell cultures tested (CB2 without urea and 3As or K12 with and without urea) (Figure 7C). CB2 is not able to oxidize ferrous iron in synthetic medium, (data not shown), which is consistent with the finding that its genome lack any detectable genes normally associated with Fe(II) oxidation. This is also true in the case of other *Thiomonas* strains (Slyemi et al., 2011). The iron precipitation observed here in AMD-impacted water in the presence of urea was therefore probably due to abiotic oxidation. In view of the dependence of the Fe(II) oxidation rate on the pH in natural waters (Sigg et al., 2006), the iron oxidation observed here may have been at least partly due to the increase in the pH observed under our experimental conditions as the result of urea degradation activity.

**Figure 8:**
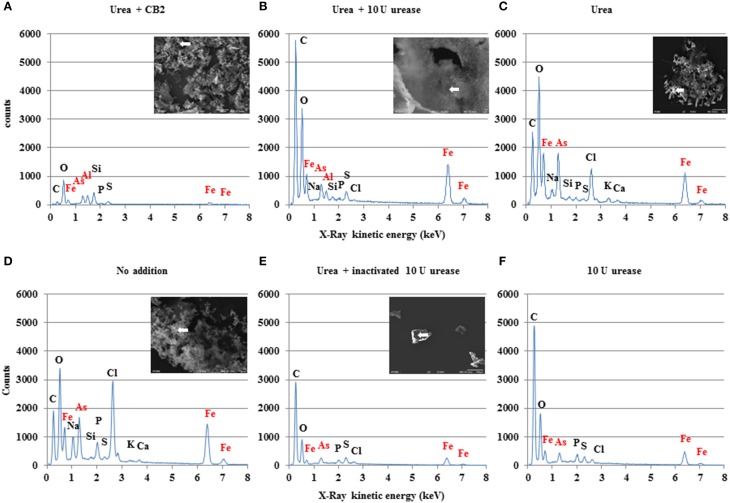
**Analysis of precipitates' composition**. SEM photographs and microanalytical X-ray graphs of precipitates. These were obtained with AMD-impacted water supplemented **(A)** with 1 g.L^−1^ urea after incubating CB2 for 4 days and **(B)** after incubating 10 U of urease in AMD-impacted water supplemented with 1 g.L^−1^ urea for 1 day, respectively. White arrows on the photographs show spots on which the X-ray microanalyses were conducted to obtain the graphs presented below. Since the magnesium X-ray K lines interfered with the arsenic L lines around 1250–1300 eV, the presence of As was confirmed by checking its K lines at higher energy levels. Silicon in the second graph and carbon in the first and third ones originate from the supports used in these studies. Pictures and graphs are representative of at least three points analyzed. These graphs show that aluminum is present in the precipitate only under the conditions where urea degradation activity was possible. Controls were also performed with **(C)** urea added alone, **(D)** no urea added **(E)** with inactivated urease, and **(F)** with urease but no urea.

**Figure 9:**
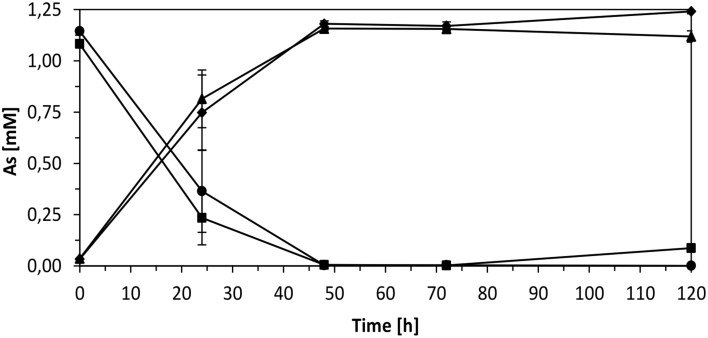
**Ability of ***Thiomonas*** strains to oxidize arsenite in m126**. Cells were grown in m126 medium supplemented with 1.33 mM arsenite, in the absence (squares and triangles) and presence of 1 g.L^−1^ urea (diamonds and circles) and concentrations of As(III) (squares and circles) and As(V) (triangles and diamonds) were measured in culture supernatants. Error bars give the standard deviations based on triplicate cultures.

**Figure 10:**
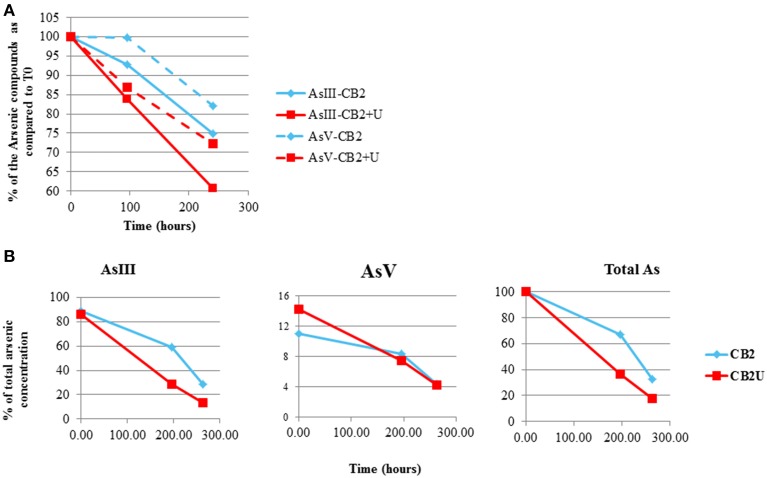
**Arsenic concentration in AMD-impacted water inoculated with CB2 in the presence and absence of urea**. As(III) and As(V) were measured by performing **(A)** GC-MS or **(B)** ICP-AES in duplicate. Arsenic concentrations are expressed as a percent of the total arsenic concentration measured at T_0_.

The pH increase observed here may have induced the co-precipitation of other metals together with Fe under these experimental conditions. Using inductively coupled plasma-mass spectrometry (ICP-MS, Table 2) and X-ray microanalysis (Figure 8), we established that in samples where urea degradation had occurred (samples inoculated with CB2 in the presence of urea), the soluble iron, arsenic, and aluminum concentrations were lower than in the other samples tested. In addition, high amounts of these elements were detected in the precipitates obtained from these samples (Table 2; Figure 8). Fe(III) precipitation probabaly causes the co-precipitation of arsenic, as previously found to occur in AMD-impacted waters (Casiot et al., 2003; Morin et al., 2003; Duquesne et al., 2008). Previous studies have shown that *Thiomonas* strains express arsenite oxidases in AMD-impacted creek waters (Bertin et al., 2011). As(III) may be oxidized into As(V), which is less soluble and precipitates with Fe(III) more efficiently than As(III) under these conditions (Casiot et al., 2003; Morin et al., 2003; Cheng et al., 2009; Maillot et al., 2013). *Thiomonas* sp. CB2 oxidizes As(III) in the presence of organic compounds (Bryan et al., 2009), and we observed in the present study that this bacterium is also able to oxidize As(III) in the presence of urea in m126 medium (Figure 9). Therefore, to test whether CB2 is able to oxidize arsenite in AMD-impacted water, the concentrations of As(III) and As(V) present in the soluble fraction were measured using ICP-AES or GC-MS. The concentrations of these two forms of arsenic decreased under all the conditions tested (Figure 10). No As(V) accumulation was observed in the soluble phase because As(V) is more efficiently adsorbed by Fe precipitates than As(III) in AMD-impacted water (Morin et al., 2003; Cheng et al., 2009; Maillot et al., 2013). These explanations are consistent with results obtained in previous studies on the geochemical processes underlying iron and arsenic solubility in AMD (Cheng et al., 2009; Klein et al., 2014). All in all, the present findings indicate that As(III) may be oxidized into As(V) and precipitated with iron more readily when urease activity is possible.

**Table 2:** **Metal content of the Precipitates and Supernatant obtained from 3As, CB2, and K12 cell cultures in AMD-impacted water**.

**Metal^a^**	**Supernatants**						**Precipitates**					
	**CB2**	**Urea + CB2**	**3AS**	**Urea + 3AS**	**K12**	**Urea + K12**	**CB2**	**Urea + CB2**	**3AS**	**Urea + 3AS**	**K12**	**Urea + K12**
Al	45.7	35.6	45.3	42.9	43.6	43.6	69.6	1.106	98.9	80.2	87.5	103
As	80.9	17.1	83.0	80.4	59.4	58.5	2.355	6.899	3660	2676	4.797	6.134
Cd	0.066	0.067	0.070	0.066	0.067	0.067	0.025	0.07	0.020	0.021	0.039	0.051
Co	0.24	0.23	0.24	0.23	0.23	0.23	0.27	0.25	0.30	0.24	0.24	0.29
Cu	0.072	0.07	0.074	0.073	0.074	0.073	0.22	0.75	0.76	0.59	0.66	0.58
Fe	882	714	874	824	847	851	5.961	20.144	11478	8488	8.189	10.224
Ni	0.47	0.46	0.46	0.46	0.47	0.45	0.64	0.73	0.81	0.64	0.62	0.67
Pb	0.2	0.075	0.22	0.20	0.065	0.066	10.3	23.9	12.6	10.4	28.4	35.3
Zn	20.5	20.2	20.8	19.5	19.9	20.1	21.2	25	27.6	21.5	21.8	26.9

a *Analysis of precipitates and supernatants was performed by ICP-MS after incubating CB2, 3As, and K12 in AMD-impacted water for 11 days in the presence and absence of 1 g.L^−1^ urea. Metal concentrations are given in mg.kg^−1^*.

Another noteworthy finding made in this study was that bacterial urea degradation activity induces aluminum precipitation. It has been established that in aluminum sulfate solutions and homogeneous solutions of aluminum acetate, sodium sulfate and urea, the reaction between water and the ammonia formed by urease activity yields hydroxide ions, which react in turn with aluminum sulfate, generating particles of aluminum basic sulfate (Simpson et al., 1998; Kara and Sahin, 2000). The AMD-impacted water used in this study certainly has a high sulfate content (Table 1). Alternatively, aluminum may precipitate in the form of aluminum oxyhydroxides, as suggested by our X-ray microanalyses (Figure 8). The pH value reached in our samples after urea degradation was slightly higher than 4.2 (Figure 6). Previous authors have indeed reported that aluminum hydroxide or microcrystalline gibbsite flocs are formed at pH values ranging from 4.2 to 4.9 in AMD and form aggregates at pH levels above 5 (Furrer et al., 2002).

**Figure 11:**
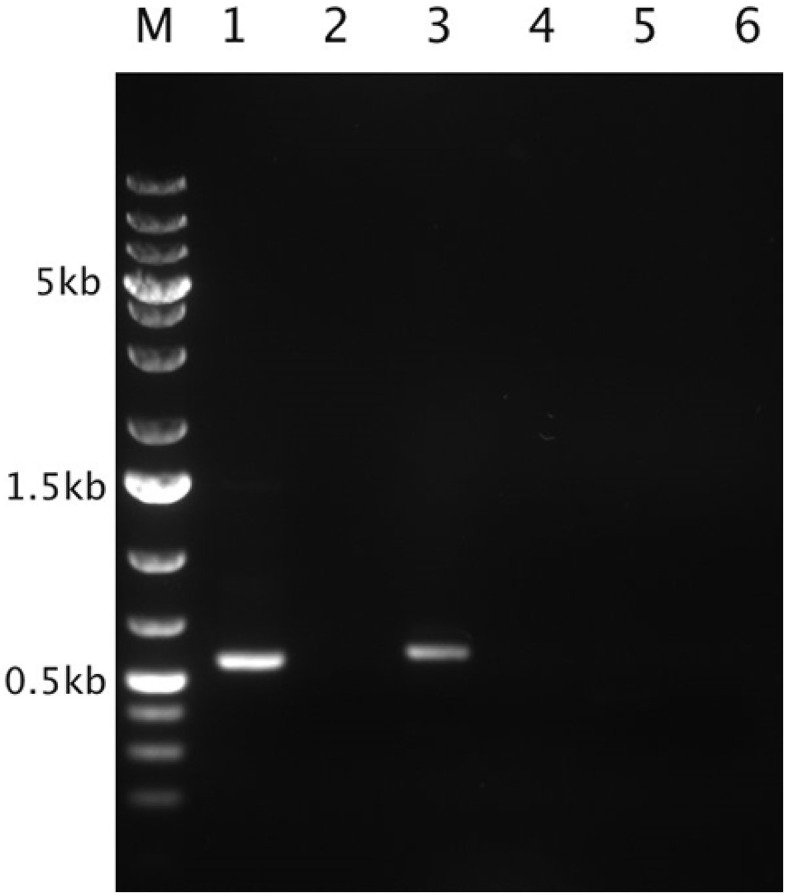
*****In situ*** expression of the ***Thiomonas*** arsenite oxidase and urease-encoding genes**. Agarose gel analysis of transcripts corresponding to *aioA, ureC*, and the gene encoding the allophanate hydrolase amplified by performing RT-PCR on the RNA extracted from the bacterial community inhabiting the Carnoulès AMD. Lane M: GeneRuler™ 1kb DNA Ladder Plus (Fermentas). Lane 2, 4, and 6: negative controls (with each gene, the same reaction was performed but without any reverse transcriptase). Lane 1: RT-PCR product in the case of *aioA* (555 bp). Lane 3: RT-PCR product in that of *ureC* (570 bp). Lane 5: RT-PCR product in that of the allophanate hydrolase gene (515 bp). The amplification products were sequenced and the sequences predicted were obtained (i. e., those of *aioA* and *ureC*).

The present data suggest that the bacterial degradation of urea may accelerate iron, arsenic, and aluminum precipitation in AMD in the presence of urea. Since urea was detected in the interstitial water of sediments from the AMD-impacted creek, it may be available to bacteria inhabiting this AMD to use for degradation purposes (Halter et al., 2012). However, the effects of urea degradation on metal precipitation were observed in this study under laboratory conditions using a higher urea concentration than that previously measured *in situ* (Halter et al., 2012). In addition, some of the *Thiomonas* strains inhabiting AMD waters are able to degrade urea whereas others are not, which suggests that urea degradation activity is not crucial to the survival of *Thiomonas* in AMD environments. In order to determine whether these biological activities are relevant *in situ*, the levels of expression of the *Thiomonas* genes encoding the arsenite oxidase and urease were therefore examined by performing RT-PCR with RNA extracted from the Reigous creek sediment community, as previously (Bertin et al., 2011). These *Thiomonas* cells extracted from the Reigous creek sediment were found to express the two genes *aioA* and *ureC* (Figure 11) responsible for the two activities promoting toxic metal precipitation, i.e., arsenite oxidation and urea degradation, respectively. These findings suggest that some of the organisms in the Carnoulès AMD community are able to produce large enough amounts of urea to promote the expression of *ure* genes *in situ*. CB2 may benefit indirectly from microbial activities in microscale environmental niches where the urea concentrations may be higher than those previously measured in the macroscale environment. This bacterium is known to be able to form biofilms, for instance (Marchal et al., 2011): other organisms surrounding *Thiomonas* cells in biofilms may provide metabolites such as urea or make the environmental conditions more suitable for CB2 survival and growth. These organisms may in turn benefit from the urea degradation and arsenite oxidation activities performed by *Thiomonas*, since both activities accelerate the precipitation of toxic metals. The urea degradation process promoted by *Thiomonas* may therefore contribute to enhancing the survival or the fitness of other microorganisms in the surrounding biofilm community, as previously suggested to occur in the case of other activities and other multi-species biofilms exposed to toxic metals (Koechler et al., 2015). Previous studies have established that the protist *E. mutabilis* present in the Carnoulès AMD is a primary producer excreting organic compounds which may be consumed by bacterial species (Bertin et al., 2011; Halter et al., 2012). Among the organic substances produced by this protist, urea was found to be excreted in synthetic medium (Halter et al., 2012). Other bacteria belonging to this community, namely “*Candidatus* Fodinabacter communificans” (Carn1 and Carn4), also carry genes involved in urea production (Bertin et al., 2011). If one of these organisms produces urea, this compound may then be degraded by *Thiomonas*. Since several organisms may be responsible of urea production, some of which have not yet been grown and studied *in vitro*, a new experimental approach will be required to identify the source(s) of urea and the relevance of its degradation to the survival of *Thiomonas* in AMDs. Generally speaking, the results presented here confirm the complexity of these processes, which probably involve interactions both between *Thiomonas* and the urea producers (*E. mutabilis* or other less well documented bacteria such as “*Candidatus* Fodinabacter communificans”) and with other mechanisms (such as arsenite oxidation). In conclusion, it emerges that several closely related *Thiomonas* strains co-exist in the Carnoulès AMD waters, which have different abilities in this toxic environment. Recent studies have shown that several closely-related bacteria co-existing in AMD biofilms express different proteins and therefore play distinct ecological roles (Denef et al., 2010). Further studies on this fine-scale heterogeneity and the interactions occurring between *Thiomonas* strains and other members of the AMD community will now be required in order to understand more clearly how these communities function and survive in these highly toxic ecosystems.

## Author contributions

JF, JA, DaH, MM, DH, RL, CC, PB, and FA designed the research project; JF, JA, DaH, CC, DH, SK, RL, MM, FS, and FA performed the research; VB, FP, and JaF contributed new reagents/analytical tools; JF, JA, DaH, CC, RL, DH, MM, FP, PB, and FA analyzed the data; and JF, PB, FP, and FA wrote the paper.

### Conflict of interest statement

The authors declare that the research was conducted in the absence of any commercial or financial relationships that could be construed as a potential conflict of interest.
