# Supplementary material for: Thiomonas sp. CB2 is able to degrade urea and promote toxic metal precipitation in acid mine drainage waters supplemented with urea
Source: Front Microbiol. 2015 Sep 28;6:993. doi: 10.3389/fmicb.2015.00993 (PMC4585258; doi:10.3389/fmicb.2015.00993)
Supplement: Supplementary file 2 [file DataSheet1.DOCX]

**Legend of supplementary Figures**

**Supplementary Figure 1. Urea degradation activity in *Thiomonas* strains.** After growth of cell cultures in m126 supplemented without or with 0.0005, 1, 2 or 5 g.L^-1^ urea, urea degradation activity was detected using Bromo-cresol purple as described in Material and Methods after incubation at room temperature for 15 min. The purple color indicates urea degradation. In the negative control where no urea was added to the BCP reagent, the orange color stands for no activity observed (as indicated by "urease test – urea"). Urea degradation affected the pH of the media when cells were incubated in the synthetic medium m126 supplemented with urea..
