# Supplementary figures and images for: Thiomonas sp. CB2 is able to degrade urea and promote toxic metal precipitation in acid mine drainage waters supplemented with urea
Source: Front Microbiol. 2015 Sep 28;6:993. doi: 10.3389/fmicb.2015.00993 (PMC4585258; doi:10.3389/fmicb.2015.00993)

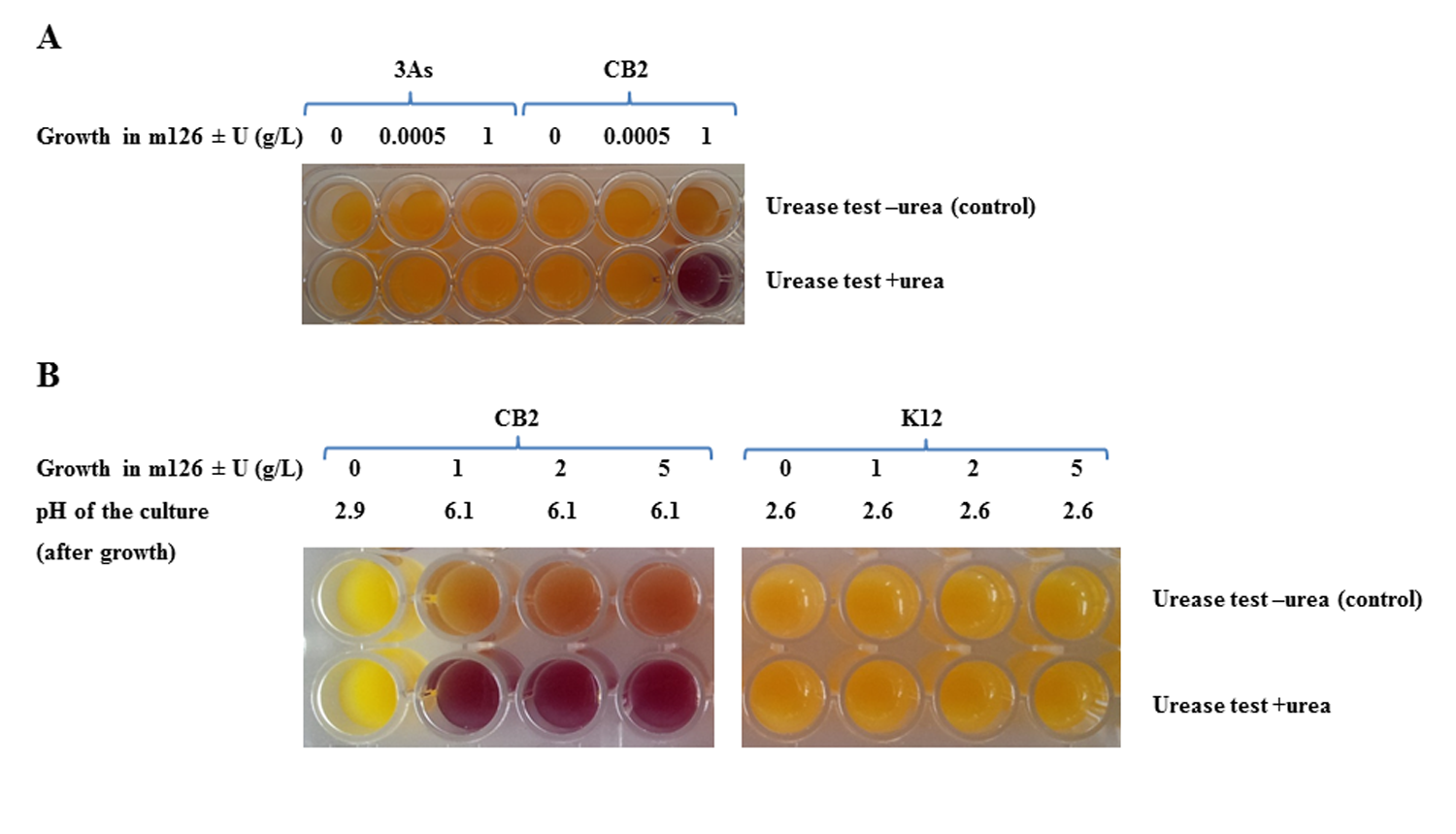

Supplement: Supplementary file 3 [file Image1.TIF]
